# The Regulatory Role of CTCF in IL6 Gene Transcription Assessed in Breast Cancer Cell Lines

**DOI:** 10.3390/ph18030305

**Published:** 2025-02-23

**Authors:** Angel Francisco Pacheco-Hernandez, Itayesitl Rodriguez-Ramos, Karla Vazquez-Santillan, Ricardo Valle-Rios, Marco Velasco-Velázquez, Guillermo Aquino-Jarquin, Gustavo Ulises Martínez-Ruiz

**Affiliations:** 1 Research Division, School of Medicine, Universidad Nacional Autónoma de México, Mexico City 04510, Mexico; francisco_p_h@outlook.com (A.F.P.-H.); ittayesitl@gmail.com (I.R.-R.); vallerios@unam.mx (R.V.-R.); 2 Precision Medicine Innovation Laboratory, National Institute of Genomic Medicine, Mexico City 14610, Mexico; kivazquez@inmegen.gob.mx; 3 Immunology and Proteomics Research Laboratory, ‘Federico Gómez’ Children’s Hospital of Mexico, Mexico City 06720, Mexico; 4 Department of Pharmacology, School of Medicine, Universidad Nacional Autónoma de México, Mexico City 04510, Mexico; marcovelasco@unam.mx; 5 RNA Biology and Genome Editing Section, Genomics, Genetics, and Bioinformatics Research Laboratory, ‘Federico Gómez’ Children’s Hospital of Mexico, Mexico City 06720, Mexico; guillaqui@himfg.edu.mx; 6 Immunobiology and Oncology Research Laboratory, ‘Federico Gómez’ Children’s Hospital of Mexico, Mexico City 06720, Mexico

**Keywords:** interleukin-6, CTCF, breast cancer, tamoxifen, methylation, gene regulation

## Abstract

**Background:** Breast cancer (BrCa) patients with tumors expressing high interleukin-6 (IL6) levels have poor clinical outcomes. In BrCa, altered occupancy of CCCTC-binding factor (CTCF) within its DNA binding sites deregulates the expression of its targeted genes. In this study, we investigated whether CTCF contributes to the altered *IL6* expression in BrCa. **Methods/Results:** We performed CTCF gain- and loss-of-function assays in BrCa cell lines and observed an inverse correlation between *CTCF* and *IL6* expression levels. To understand how CTCF negatively regulates *IL6* gene expression, we performed luciferase gene reporter assays, site-directed mutagenesis assays, and chromatin immunoprecipitation assays. Our findings revealed that CTCF interacted with the *IL6* promoter, a form of regulation disrupted in a CpG methylation-independent fashion in MDA-MB-231 and Tamoxifen-resistant MCF7 cells. Data from TCGA and GEO databases allowed us to explore the clinical implications of our results. An inverse correlation between *CTCF* and *IL6* expression levels was seen in disease-free survival BrCa patients but not in patients who experienced cancer recurrence. **Conclusions:** Our findings provide evidence that the CTCF-mediated negative regulation of the *IL6* gene is lost in highly tumorigenic BrCa cells.

## 1. Introduction

Interleukin-6 (IL6) modulates diverse physiological processes, such as inflammation, differentiation, and cell growth [1,2]. Aberrant IL6 expression has been reported in several diseases, including cancer [2,3]. In breast cancer (BrCa), IL6 expression correlates with worse clinical scenarios, such as metastasis and antitumoral therapy resistance [4,5,6]. BrCa’s tumors are classified into the following categories: Estrogen Receptor (ER+) which includes luminal A and B subtypes, human epidermal growth factor (HER2)-enriched, and triple-negative BrCa (TNBC), the last of which encompasses the basal-like and claudin-low subtypes [7].

The large majority (70–80%) of BrCa tumors are of the ER+ subtype. Despite the availability of antitumoral ER-directed therapies [8], 30–40% of patients are refractory to them, increasing the likelihood of developing metastasis [8,9]. Tumors resistant to ER-directed antitumoral therapies exhibit higher IL6 expression levels [10,11]. In HER2-enriched BrCa, IL6 also promotes tumor progression [12,13]. TNBC is the most lethal BrCa subtype and has limited antitumor therapy options. Inhibiting *IL6* expression or blocking IL6 with antibodies decreases the TNBC’s malignancy [14,15,16,17]. Overall, this evidence highlights that IL6 provides survival advantages to BrCa cells under the selective pressures. Thus, identifying cell-intrinsic regulators of *IL6* gene expression in BrCa may help to conceptualize new antitumoral therapies.

CTCF regulates the tridimensional configuration of the human genome [18,19]. This multifunctional protein recognizes and binds to specific DNA binding sites, thereby controlling the gene expression profile [19,20]. Functional changes in CTCF are noticeable in BrCa and have an impact on the expression profile of its regulated genes [21,22,23].

The relationship between CTCF and *IL6* has been evaluated in several biological scenarios distinct from cancer. For example, deletion of a CpG dinucleotide located at +348 pb concerning the transcription start site (TSS) of the *IL6* gene inhibited *IL6* expression by recruiting CTCF in murine macrophages stimulated with LPS [24]. This finding suggests that CTCF represses *IL6* transcription. Intriguingly, in vitro-differentiated macrophages from isolated bone marrow cells from conditionally CTCF-deficient mice did not show alterations in *IL6* expression upon stimulation with LPS [25]. It is unclear whether this unresponsiveness is caused by surviving aberrant macrophages after in vitro differentiation or other causes. On the other hand, the disease severity of COVID-19 patients directly correlates with IL6 expression levels [26]. A variant haplotype encompassing the *IL6* gene seemed to protect against developing severe COVID-19 illness by reducing *IL6* expression [27]. This haplotype harbors an SNP located within intron 2 of the *IL6* gene that inhibited the binding of CTCF, which was required to transcriptionally induce the *IL6 antisense RNA1* (*IL6-AS1*) gene expression upon a variety of stimuli [27]. *IL6-AS1* gene transcribes a long non-coding RNA from the complementary strand of the *IL6* gene and overlaps 67 pb with the *IL6* gene [28]. As *IL6-AS1* and *IL6* gene expressions correlated with each other among the stimuli analyzed, this SNP was proposed to regulate *IL6* expression [27]. However, IL6-AS1 may both protect *IL6* mRNA from degradation and recruit transcriptional activators to the *IL6* promoter [28]. Thus, whether the CTCF binding in intron 2 of the *IL6* gene directly controls IL6 expression is currently unknown. Even though the regulatory role of CTCF in the *IL6* might be cell-type specific, the current knowledge about this relationship is unknown in BrCa.

Herein, we investigated whether CTCF might regulate *IL6* expression in BrCa cells. Our experiments, involving both CTCF gain- and loss-of-function assays, revealed an inverse correlation between *CTCF* and *IL6* gene expression in BrCa cells. Using gene-reporter assays, site-directed mutagenesis, and chromatin immunoprecipitation (ChIP) assays, we demonstrated that CTCF directly interacts with the *IL6* promoter in MCF7 cells, which exhibit low *IL6* gene expression. Conversely, we observed a loss of CTCF interaction with the *IL6* promoter in IL6-high-expressing BrCa cells, such as in MDA-MB-231 and Tamoxifen-resistant MCF7. Since CTCF interactions with some of its DNA binding sites are methylation-sensitive, we examined the relationship between CTCF deposition and DNA methylation and found no evident correlation. Finally, we addressed the clinical relevance of our results by analyzing publicly available databases. We observed a significant inverse correlation between *CTCF* and *IL6* gene expression levels in BrCa samples from patients with disease-free survival but not in those with cancer recurrence. In conclusion, our results indicate that CTCF restrains *IL6* expression by interacting with its promoter, a regulation lost in highly tumorigenic cells. This regulatory relationship is clinically observable through data retrieved from the GEO and TCGA database.

## 2. Results

### 2.1. CTCF Inhibits IL6 Gene Transcription in BC Cell Lines

**Figure 1:**
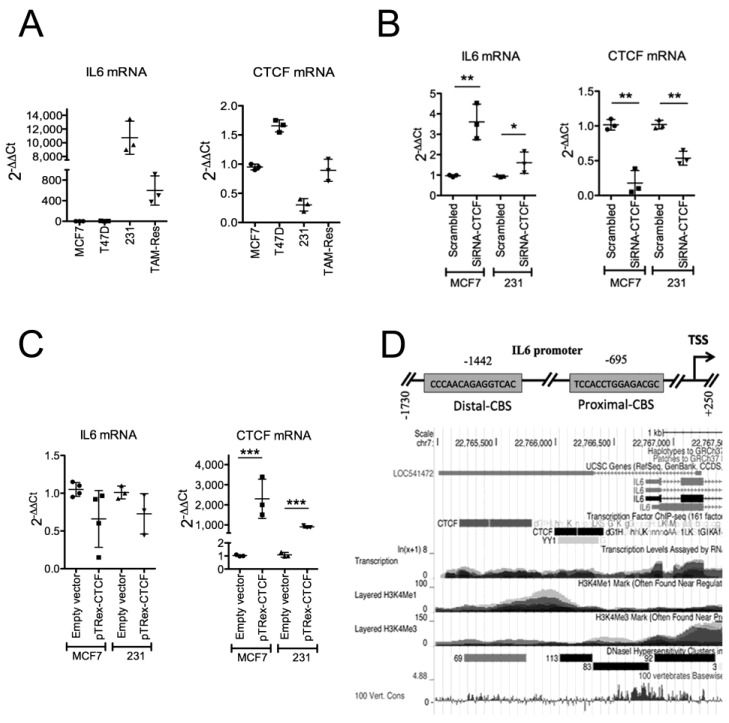
CTCF negatively regulates *IL6* gene transcription in BrCa cell lines. (**A**) *IL6* and *CTCF* expression levels in MCF7, T47D, MDA-MB-231 (231), and Tamoxifen-resistant MCF7 cells (Tam-res) were determined by RT-qPCR. (**B**) MCF7 and MDA-MB-231 (231) cells were transiently transfected with specific CTCF siRNAs or control-siRNAs (scrambled). RT-qPCR assays were performed to determine the *IL6* and *CTCF* gene expression levels. (**C**) MCF7 and MDA-MB-231 (231) cells were transiently transfected with the plasmid pTRex–CTCF or pTRex empty vector and the expressions of *IL6* and the *CTCF* gene were determined by RT-qPCR assays. Graphs A-C show the standard deviation from at least three independent experiments. (**D**) Upper panel, the CTCF binding sites located in the *IL6* promoter sequence are depicted. Lower panel, CTCF ChIP-seq data for the *IL6* promoter shown were retrieved from the UCSC genome browser. * *p* < 0.05, ** *p* < 0.01, and *** *p* < 0.001.

**Figure A1:**
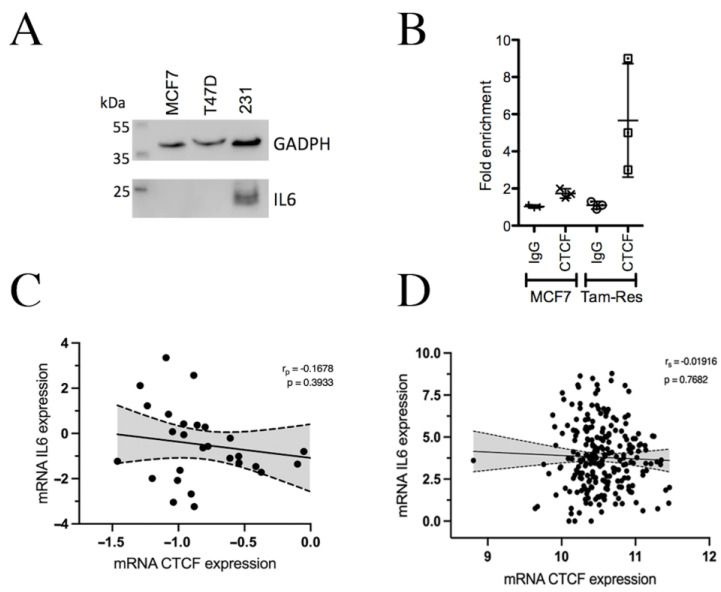
(**A**) Protein levels of IL6 and GAPDH were assessed using Western blot assays on total protein extracts from MCF7, T47D, and MDA-MB-231 cells. This figure represents two independent experiments. (**B**) YY1 ChIP-qPCR assays were performed in both Tamoxifen-resistant and their parental MCF7 cells. IgG antibody was used as a control for ChIP-qPCR assays. The standard deviation from three independent experiments is shown (**C**). Data generated by Xia and colleagues [42] were retrieved from the GEO [41] database to obtain both IL6 and CTCF expression levels of samples from ductal breast cancer patients who experienced cancer recurrence. BrCa patient data retrieved from The Cancer Genome Atlas (TCGA) database were divided into quartiles according to their disease-free interval (DFI). (**D**). The expression values of IL6 and CTCF genes from BrCa patients within the first quartile (patients who were DFI in less than 436 d) did not show any statistical correlation. r_p_, Pearson’s correlation analyses; r_s_, Spearman’s correlation analyses; *p*, *p*-value.

We quantified the *IL6* and *CTCF* gene expression levels in multiple BrCa cell lines, including ER+ (MCF7 and T47D), Tamoxifen-resistant MCF7, and TNBC (MDA-MB-231), at the mRNA level by qPCR (Figure 1A). As previously reported [29,30], MDA-MB-231 cells exhibited the highest IL6 protein expression levels compared with either MCF7 or T47D cells (Figure A1(A)). To inspect the participation of CTCF in the transcriptional regulation of the *IL6* gene, we downregulated *CTCF* mRNA expression by transitorily transfecting specific siRNAs against *CTCF* mRNA in both MCF7 and MDA-MB-231 cancer cells. As expected, *CTCF* mRNA expression was effectively downregulated in each cell line (Figure 1B, left). We found higher expression levels of *IL6* in cells transfected with CTCF siRNAs than those transfected with control (scrambled) siRNAs (Figure 1B, right). Further, we conducted gain-of-function assays for CTCF by transiently transfecting MCF7 and MDA-MB-231 cells with a plasmid containing the open reading frame of the *CTCF* gene [31]. Cells ectopically expressing CTCF (Figure 1C, left) showed a tendency to downregulate *IL6* mRNA expression (Figure 1C, right). This outcome aligns with our expectation, as these cells exhibited constitutive CTCF expression (Figure 1A, left). Therefore, these assays suggest a negative regulatory role of CTCF in the *IL6* gene expression.

### 2.2. CTCF Binding Sites Located in the IL6 Promoter Restrain IL6 Expression

CTCF regulates the transcriptional profile by multiple mechanisms, relying on its ability to interact with its DNA binding sites [18,19]. Because we observed that CTCF negatively regulates *IL6* expression in CTCF gain- and loss-of-function assays, we evaluated the binding of CTCF with the *IL6* promoter and its functional consequences.

**Figure 2:**
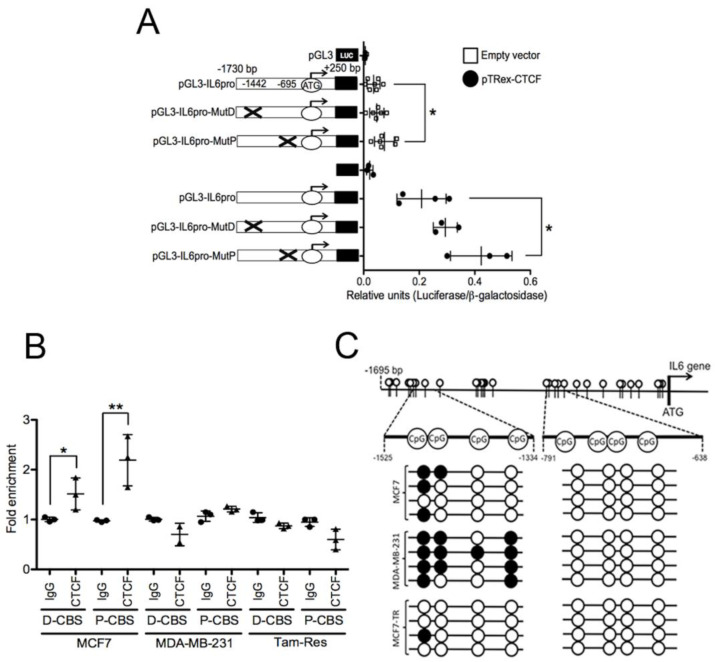
CTCF restrains *IL6* expression by interacting with the *IL6* promoter sequence. (**A**) Reporter gene assays in MCF7 cells transitorily transfected with the *IL6* gene promoter sequence into a pGL3 vector (pGL3-IL6pro) or its mutant versions harboring deletions of the CTCF binding sites. These gene–reporter plasmids were co-transfected with either the pTRex–CTCF plasmid (black circles) or the pTRex empty vector (white boxes). (**B**) CTCF ChIP qPCR assays performed in parental MCF7, MDA-MB-231, and Tamoxifen-resistant (Tam-Res) MCF7 cells. (**C**) The methylation status of the CBS in the *IL6* promoter was determined by bisulfite genomic sequencing in DNA extracted from MCF7, MDA-MB-231 cells, and Tamoxifen-resistant cells. Black and white circles indicate methylated and unmethylated CpG dinucleotides, respectively. The standard deviation from at least three independent experiments is shown. CBS, CTCF binding site; P-CBS, Proximal-CBS; D-CBS, Distal-CBS. * *p* < 0.05 and ** *p* < 0.01.

We bioinformatically identified two putative CTCF binding sites (CBSs) in the *IL6* promoter by using the JASPAR’s position weight matrices [32,33]. These CBS are at −1442 bp (Distal-CBS) and −695 bp (Proximal-CBS) concerning the transcription start site (TSS) of the *IL6* gene (Figure 1D, upper panel). We consulted CTCF ChIP-Seq data deposited in the UCSC Genome Browser [34] and observed that two CTCF-enriched genomic segments in the *IL6* gene overlapped with the CBS identified (Figure 1D, lower panel). To assess the regulatory role of these CBSs on *IL6* transcription, we performed plasmid-based gene-reporter assays. Thus, we cloned the *IL6* promoter into a luciferase–reporter plasmid (pGL3-IL6pro) and deleted either the Distal- or the Proximal-CBS, generating the pGL3-IL6pro-MutDis and pGL3-IL6pro-MutPro plasmid constructs, respectively. We determined the gene reporter levels in transitorily transfected MCF7 cells. The deletion of any of CBSs in the *IL6* promoter induced higher normalized luciferase levels compared with cells transfected with the wild-type *IL6* promoter gene-reporter plasmid, being significant for the proximal CBS (Figure 2A). We observed the same trend in cells harboring the ectopic expression of CTCF (Figure 2A). Thus, CTCF requires the proximal CBS in the *IL6* promoter to restrain IL6 expression.

### 2.3. CTCF Restrains IL6 Transcription by Interacting with IL6 Promoter

We observed that the ectopic modulation of CTCF levels inversely correlated with the expression of the *IL6* gene (Figure 1) and that the mutation of CBSs in the *IL6* promoter enhanced *IL6* expression (Figure 2A). Therefore, we envisioned that CTCF represses *IL6* expression by binding to *IL6* promoter. As MCF7 and MDA-MB-231 cells express low and high *IL6* expression, respectively (Figure 1), we inspected the differences in CTCF binding over the *IL6* promoter in these cell lines. By the CTCF ChIP-qPCR assays, we identified that CTCF binds to the *IL6* promoter in MCF7 cells but not in MDA-MD-231 cells (Figure 2B). Our results suggest that CTCF restrains *IL6* expression by binding to the *IL6* promoter, mainly in the proximal CBS, in MCF7 cells, which have been featured by their low tumorigenic potential.

### 2.4. Tamoxifen-Resistant Breast Cancer Cells Exhibit Higher IL6 Expression and Loss of CTCF Binding in the IL6 Promoter

We were interested in defining CTCF’s possible regulatory role on *IL6* expression in a therapy-resistant model, because IL6 expression leads to hormonal therapy resistance in BrCa [8,30]. Thus, we performed CTCF ChIP-qPCR assays in a tamoxifen-resistant cell line with high *IL6* expression levels (Figure 1A). CTCF did not interact with the *IL6* promoter in the Tamoxifen-resistant MCF7 cells (Figure 2B), an opposite observation to that for the parental MCF7 cells. Similarly to Tamoxifen-resistant cells, MDA-MB-231 lost CTCF binding over the *IL6* promoter, highlighting the repressing effect of CTCF on the *IL6* gene.

To assess the specificity of CTCF binding to the *IL6* promoter in Tamoxifen-resistant cells, we further inspected whether deposition of other TFs over the *IL6* promoter would correlate with its *IL6* transcriptional expression. Inspection of the YY1 ChIP-seq data available in the Genome Browser server [34] revealed a YY1 binding site near the proximal CBS on the *IL6* promoter (Figure 1A). YY1 ChIP-qPCR assays demonstrated the association of YY1 with the *IL6* promoter in Tamoxifen-resistant MCF7 but not in their parental cells (Figure A1(B)). Previous studies have shown that YY1 regulates IL6 expression in a variety of conditions, including LPS-stimulated BV2 microglial cells [35], and in vivo models for rheumatoid arthritis [36] and prostate cancer [37]. Given that YY1 plays a role in defining transcription profiles by forming DNA loops in CTCF-flanked genomic zones [38,39], we hypothesize that CTCF and YY1 might regulate *IL6* transcription by modulating its chromatin configuration, which is worth future investigation.

CTCF interacts in a methylation-sensitive fashion in nearly 40% of its DNA binding sites [40]. Therefore, we inspected whether the CpG dinucleotide methylation profile would explain changes in CTCF deposition over the *IL6* promoter among the cell lines analyzed. We observed a not obvious correlation between the methylation profile in either the Proximal- or Distal-CBS in the *IL6* promoter across the cell lines analyzed with CTCF deposition (Figure 2C).

Taken together, these results highlight the restraining effect of CTCF on *IL6* gene transcription though its interaction with the *IL6* promoter, which is not present in highly tumorigenic cells such as MDA-MB-231 and Tamoxifen-resistant cells.

### 2.5. CTCF and IL6 Expression Levels Are Inversely Correlated in a Subset of Breast Cancer Patients

**Figure 3:**
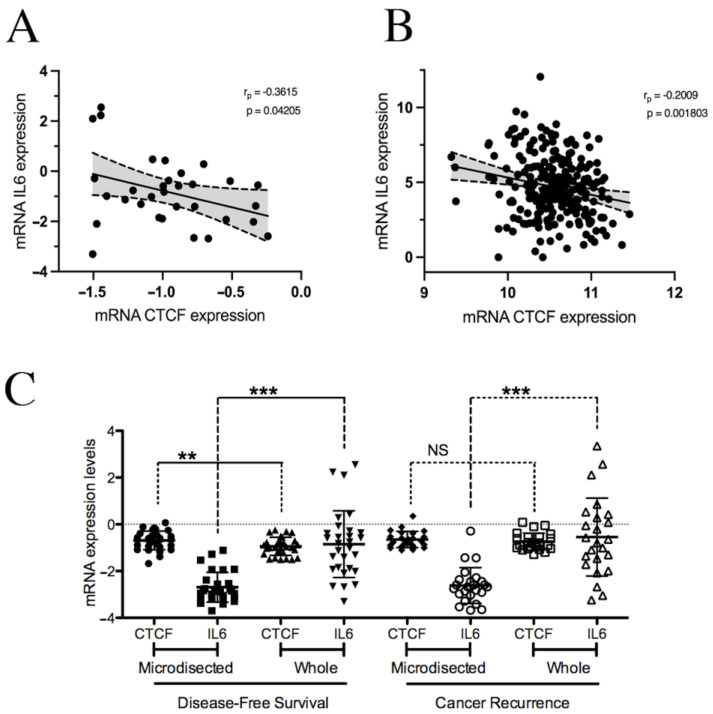
*IL6* and *CTCF* gene expression in breast cancer patient samples. (**A**) *IL6* and *CTCF* expression levels from data generated by Xia et al. [42] accessed by using the GEO database [41]. (**B**) *IL6* and *CTCF* gene expression in BrCa patient data retrieved from TCGA database [43]. The data shown correspond to patients within the last quartile (more that 1562 d), as classified by their DFI. (**C**) Analysis of *IL6* and *CTCF* expression in laser-captured microdissection samples (microdissected) compared with whole breast tumor samples. The standard deviation is shown. NS, not significant; r_p_, Pearson’s correlation; *p*, *p*-value. ** *p* < 0.01 and *** *p* < 0.001.

To delve into the clinical significance of the regulatory role of CTCF in *IL6* expression uncovered here, we retrieved gene expression data using the GEO database [41] generated by Xiao-Jun and colleagues, who performed microarray experiments from ER-positive ductal BrCa patient samples. After standard breast surgery and following radiation, these patients were treated with Tamoxifen as an adjuvant therapy for five years [42]. We found a significant negative correlation between *CTCF* and *IL6* gene expression levels in patients with DFS (disease-free survival) at the time of analysis (Figure 3A). This shows that CTCF restrains *IL6* gene expression in tumors sensitive to antiestrogenic therapy, correlating with our in vitro findings. On the other hand, we did not observe any correlation between *IL6* and *CTCF* expression levels in patients who underwent cancer recurrence (Figure A1(C)). In concordance with this, our results, obtained from cellular models of aggressive tumors (MDA-MB-231 and Tamoxifen-resistant MCF7 cells), showed no evident regulatory effect of CTCF on *IL6* expression.

Our observations were extended analyzing BrCa data from TCGA. Patients were split into quartiles using their DFI. The *IL6* and *CTCF* expression levels exhibited a statistically inverse correlation in the highest quartile (patients with a DFI > 1562 d; Figure 3B). In contrast, no correlation was found in patients within the lowest quartile, who experienced tumor recurrence in less than 436 days (Figure A1(D)). Thus, *CTCF* expression and *IL6* expression were inversely correlated in BrCa patients with a good response, suggesting that this relationship may be relevant in a subset of patients with unaggressive tumors.

We also analyzed the co-expression of *CTCF* and *IL6* in different tumor regions using data from Xiao-Jun Ma and collaborators [42]. *IL6* gene expression was higher in the whole tumor tissue than in cancer cell-enriched samples obtained by laser capture microdissection (LCM) (Figure 3C). This observation suggests the need for further investigation into whether the CTCF–IL6 axis is also present in cancer-associated cells such as corrupted macrophages and fibroblasts [44,45,46].

## 3. Discussion

Aberrant IL6 expression is associated with poor clinical outcomes in BrCa, such as metastasis and resistance to therapy [3,15,30,47,48]. On the other hand, functional alterations of CTCF are observed in BrCa [21,22,23], dysregulating the expression of its targeted genes, such as *XAF1* [31], *Bax* [49], and *HOXA10* [50]. Thus, we analyzed the possible regulation of CTCF on *IL6* expression in BrCa cells. After performing in vitro CTCF gain- and loss-of-function assays, we demonstrated an inverse correlation between *CTCF* and *IL6* expression levels. Furthermore, we identified and validated two CTCF binding sites in the *IL6* promoter that could directly regulate *IL6* expression, based on previous investigations [51]. Remarkably, CTCF inhibited *IL6* expression by interacting with the *IL6* promoter in ER + MCF7 cells, which display low tumorigenic and metastatic potential and are sensitive to antiestrogenic therapy [8,52]. Since IL6 expression drives resistance to anticancer therapy in BrCa [30,53,54], we generated a Tamoxifen-resistant cell line that, as well as MDA-MB-231 cells, exhibited higher *IL6* expression and no deposition of CTCF over the *IL6* promoter. This is in concordance with a previous report showing reduced CTCF binding to the active re-compartmentalized genomic areas in Tamoxifen-resistant cells [55]. Finally, we found a statistically significant inverse correlation between *CTCF* and *IL6* expression levels in BrCa tissues from patients with good prognoses, but not in those with cancer recurrence. These findings further strengthen our in vitro results, showing that the negative regulation of CTCF over *IL6* expression was present in clinical samples. Overall, we provide evidence that CTCF retrains *IL6* expression by interacting with the *IL6* promoter, and this regulation is broken in highly tumorigenic cells and in aggressive BrCa tumors.

Future efforts should focus on delineating the mechanisms driving CTCF’s interaction with the *IL6* promoter. For example, point mutations of the *IL6* promoter’s CTCF binding sites might explain the loss of CTCF binding. However, there are multiple reports supporting the role of post-translational modifications to CTCF in regulating its ability to interact with its CBS and its nuclear residency [23,56,57]. Interestingly, O-GlcNAcylated CTCF levels increased in embryonic stem cells, which was required for maintaining stemness as well as the 3D chromatin configuration by modulating chromatin loop formation instead of modifying A/B compartments [58]. Because differences in cancer stem cell frequencies exist between highly and low-tumorigenic BrCa cells [59], exploring whether O-GlcNAcylated CTCF regulates the 3D chromatin shape in the *IL6* gene across BrCa cells with different stem cell proportions warrants further investigation.

Exhausted T cells in tumors cannot eliminate malignant cells because they release dramatically fewer effector cytokines, exhibit limited cytolytic activity, and express inhibitory receptors such as programmed cell death protein 1 (PD1). Interestingly, IL6 increased PD1 gene expression in TCR-stimulated CD8^+^ T cells [60], suggesting its possible role in T cell exhaustion. Concordantly, intra-tumoral IL6 expression increases the content of PD1^+^ T cells [61,62] by restraining the conversion of CD8^+^ T cells into cytotoxic fate and causing them to polarize in an exhaustion state in an IL6R/STAT3-dependent manner [63]. In agreement with these results, circulating CD8^+^ T cells from high-IL6-producing cancer patients showed a transcriptional profile that exhibited their hypofunctional state [63]. Therefore, determining the role of the CTCF–IL6 axis in the niche of T cell exhaustion might lead to the conceptualization of new therapeutic options.

## 4. Materials and Methods

### 4.1. Cell Culture

MCF7, T47D, and MDA-MB-231 cancer cell lines were purchased from the American Type Culture Collection (ATCC, Manassas, VA, USA). MCF7 and MDA-MB-231 were maintained in Dulbecco’s Modified Eagle medium, without phenol red, and supplemented with 5 and 10% of fetal bovine serum (FBS), respectively. T47D cells were maintained in RPMI Advance medium supplemented with 5% SFB. All culture media were also supplemented with L-glutamine and Non-Essential Amino Acids. The cells were grown in a humidified incubator at 37 °C with 5% CO_2_. 4-hydroxytamoxifen (Tamoxifen) was purchased from Sigma-Aldrich (St Louis, MO, USA). As previously reported [64], the Tamoxifen-resistant cancer cell line was generated from MCF7 cells. Briefly, MCF7 cells were continuously exposed to increasing concentrations of Tamoxifen until they reached a final concentration of 10^−7^ M. Cell cultures were passaged by trypsinization when they exhibited 70% of confluency. The medium was replaced every four days with a fresh medium containing Tamoxifen. We designated Tam-Res cells as MCF7 cells that had grown after 4 months in a medium containing Tamoxifen at 10^−7^ M.

### 4.2. RT-qPCR Assays

Total RNA was extracted from the BrCa cell lines in this study by using TRIzol reagent (Invitrogen, Waltham, MA, USA), and it was subjected to reverse transcription using the Superscript III kit (Invitrogen, Waltham, MA, USA) to generate cDNA from 500 ng of total RNA, following the manufacturer’s instructions. Then, qPCR assays were performed using 1 μL of cDNA and the SYBR Green Master Mix (Applied Biosystems, Foster city, CA, USA). The set of primers used in this work were as follows: 5′- cagcctcaagatcatcagcaatg-3′ (GADPH-Sense), 5-catgagtccttccacgataccaa-3′ (GADPH-Antisense), 5’-tgcggaaagtgaacccatgata-3′ (CTCF-Sense), 5’-cccttgttctagtgtctccacc-3′ (CTCF-Antisense), 5′- cttggtgaggaagtttcagaaca-3′ (IL6-Sense), 5’-acgcacatggacactatgtagaa-3′ (IL6-Antisense), 5’-ttgctgacctgctggattacat -3′ (HPRT1-Sense) and 5’-cccctgttgactggtcattaca-3′ (HPRT1-Antisense). The thermal cycling conditions for PCR reactions were 95 °C for a 10 min denaturation step, followed by 40 PCR cycles of [95 °C (30 s) and 59 °C (60 s)].

### 4.3. Plasmid Constructs

The promoter sequence of *IL6* gene encompassing nucleotides -1730 to +250 bp around its transcription start site was amplified using Platinum Pfx DNA Polymerase (Thermo Scientific, Waltham, MA, USA) with the following primers: 5’-aaccggttcacagtgcacggctg-3′ and 5’-agaattctggggcagggaaggcag-3′; these primers contain AgeI and EcoRI restriction enzyme sites (indicated in bold and underline), respectively. The PCR product was cloned into pCR2.1 (Thermo Scientific, Waltham, MA, USA). It was subcloned into the pGL3 plasmid by a directional cloning strategy using the restriction enzymes mentioned above, thus generating the plasmid construct pGL3–IL6pro. The putative CTCF binding sites were deleted using the site-directed mutagenesis procedure. Briefly, the deletions were induced by PCR reactions using the following primers for the deletion of the distant CTCF binding site (respect to the TSS of *IL6* gene): 5’-tgcacgaaacaaaacttgagtaaagcttttatcgatcttgaagagatct-3′ (Del1-Sense) and 5′- agatctcttcaagatcgataaaagctttactcaagttttgtttcgtgca-3′ (Del1-Antisense); for deletion of the Proximal CTCF binding site, the following were used: 5′- gcaaaaaggagtcacacaccggtaactgcacgaaatttga-3′ (Del2-Sense) and 5′- tcaaatttcgtgcagttaccggtgtgtgactcctttttgc-3′ (Del2-Antisense). Briefly, 25 ng of the pGL3-IL6pro plasmid was used as a template for these PCR reactions. Then, the PCR products were digested with Dpn1 restriction enzyme at 37 °C for 2 h. Subsequently, *E. coli* DH5α bacteria were transformed with the digested PCR products. All plasmids (pGL3-IL6pro, pGL3-IL6pro-MutD, and pGL3-IL6pro-MutP) were verified by capillary sequencing. The plasmid construct with the coding sequence of CTCF was generated previously [31].

### 4.4. Plasmid Transfections

Briefly, 2.5 × 10^5^ MCF7 cells were seeded in 35 mm plates. After 18 h, the cells were co-transfected with 1.8 μg of the plasmid with the IL6 promoter sequence (pGL3-IL6pro, pGL3-IL6pro-Del1 or pGL3-IL6-pro-Del2) and 0.2 μg of pCMVSport-βGal (Thermo Scientific, Waltham, MA, USA) using Lipofectamine 2000 (Invitrogen, Waltham, MA, USA), following the manufacturer’s instructions. At 24 h post-transfection, the cells were lysed to measure both β-galactosidase and luciferase activities using Luminescent β-galactosidase Detection Kit II (Takara Bio Inc., Kusatsu, Japan) and the Luciferase assay system (Promega, Madison, WI, USA).

### 4.5. siRNA Knockdown

Then, 2.5 × 10^5^ MCF7 or MDA-MB-231 cells were seeded in 35 mm plates. After 18 h, the cells were transfected with human CTCF small interfering RNAs (TriFECTa RNAi Kit; Integrated DNA technologies, Coralville, IA, USA) or a scrambled sequence at 0.1μM by RNAiMax (Invitrogen, Waltham, MA, USA), following the manufacturer’s instructions. At 24 h post-transfection, RNA was isolated for RT-qPCR assays.

### 4.6. Bisulfite DNA Sequencing

DNA was extracted from MCF7, MDA-MB-231, or the Tamoxifen-resistant cells using an unniPREP DNA mini kit (Analytik Jena AG, Jena, Germany). An amount of 1.5 μg of DNA from each cell line was treated with the unniCONVERT Bisulphite Basic Kit (Analytik Jena AG, Jena, Germany), according to the manufacturer’s instructions, for obtaining bisulfite-converted DNA. Then, this DNA was used as a template in PCR reactions for the amplification of CpG dinucleotides overlapping with the putative CTCF binding sites in the IL6 promoter. The set of primers used for these PCR reactions were as follows: 5’-ggtagggtagtagttaatttttt-3’ (Bi-CBs-1S), 5’-ctattataaaactacctaacca-3’ (Bi-CBs-1AS), 5’-gaagaatggatgattttatttt-3’ (Bi-CBs-2S), and 5’-cacaacaccaaaacacttattt-3’ (Bi-CBs-2AS). The PCR products were cloned into the pCR2.1 vector (Thermo Scientific, Waltham, MA, USA). Methylated GpG dinucleotides were determined after the capillary sequencing of the generated plasmids.

### 4.7. Chromatin Immunoprecipitation (ChIP)

Briefly, 5 × 10^6^ MCF7, MDA-MB-231, or Tamoxifen-resistant MCF7 cells were fixed with 1% formaldehyde for crosslinking, and subsequently, the reaction was stopped by adding glycine at 0.125 M. The cells were washed three times with ice-cold phosphate-buffered saline solution and then lysed using lysis buffer (10 mM EDTA, 50 mM TRIS-HCl pH 8, 1% SDS, protease inhibitor cocktail). The lysates were sonicated using a probe sonicator to obtain chromatin with a mean length of 200 bp, and then precleared with ChIP-grade protein A/G magnetic beads (Thermo Scientific, Waltham, MA, USA). An amount of 2.5 μg of a specific antibody against CTCF (07-729; Merck Millipore, Burlington, MA, USA), YY1 (38422; Abcam, Cambridge, UK), or normal mouse IgG (10060; Merk Millipore, Burlington, MA, USA) was added to the precleared lysates and incubated at 4 °C overnight. Immunoprecipitation of protein–antibody complexes was performed by adding protein A/G magnetic beads. These complexes were washed sequentially in buffer A (20 mM Tris-HCl ph 8.0, 2 mM EDTa, 150 mM NaCl, 1%Triton X-100, 0,1% SDS), buffer B (20 mM Tris-HCl pH 8.0, 2 mM EDTA, 500 mM NaCl, 1% Triton X-100, 0. 1% SDS), buffer C (10 mM Tris-HCl pH 8.0, 1 mM EDTA, 1% sodium deoxycholate, 1% NP-40, 0.25 M LiCl) and buffer TE (10 mM Tris-HCl, 1 mM EDTA). The protein–antibody complexes were released by adding elution buffer (1% SDS, 0.1 M NaHCO_3_) at 40 °C. To reverse the crosslink, the eluted DNA was incubated with NaCl (0.2 M) at 65 °C overnight, and then with proteinase K (Qiagen, Hilden, Germany) at 45 °C for 2 h. The ChIP-enriched DNA was purified using the QIAquick PCR purification kit (Qiagen, Hilden, Germany) and amplified by qPCR using SYBR Green PCR Master Mix (Applied Biosystems, Foster city, CA, USA) with the following primers: 5’-cttgagtaaatgcccaacagagg-3′ (ChIP-CB-1S), 5’-catggtgttaccttcacaatcgg-3′ (ChIP-CB-1AS), 5’-gtggcaaaaaggagtcacacact-3′ (ChIP-CB-2S), and 5’-catctgagttcttctgtgttctgg-3′ (ChIP-CB-2AS).

### 4.8. Immunoblotting

Total protein extracts were generated by lysing cell lines in RIPA buffer (Thermo Scientific, Waltham, MA, USA) supplemented with a protease inhibitor cocktail (Promega, Madison, WI, USA). Subsequently, proteins were separated under Sodium Dodecyl-Sulfate PolyAcrylamide Gel Electrophoresis (SDS-PAGE) with 18% acrylamide. Then, the proteins were transferred to PVDF membranes, which were incubated in 5% low-fat milk in TBS-T solution (0.05% Tween 20 in TBS buffer). Membranes were incubated overnight with either anti-IL6 (D3K2N; Cell signaling technology, Danvers, MA, USA) or anti-GAPDH (D16H11; Cell signaling technology, Danvers, MA, USA) antibodies. After washing and incubating the membranes with a secondary antibody conjugated with HRP (GTX213110-01; Gene Tex, Irvine, CA, USA), proteins were detected using a chemiluminescent kit (Thermo Scientific, Waltham, MA, USA) and visualized with FUSION Solo S (Vilber, Collégien, France).

### 4.9. Analysis of Data Retrieved from the Gene Expression Omnibus (GEO) or the Cancer Genome Atlas (TCGA) Databases

Data were retrieved from the Gene Expression Omnibus (GEO) database from the accession numbers GSE1378 and GSE1379, these data having been deposited by Xiao-Jun Ma et al. [42]. We did not discard any data from the available gene expression results from ductal breast cancer patient samples. BrCa patient sample data (Breast Invasive Carcinoma, Firehose Legacy) were downloaded from TCGA database by using the Xena Browser [43]. The BrCa patients were grouped into quartiles based on their disease-free interval (DFI), and their IL6 and CTCF gene expression values were retrieved. The first and the last quartiles encompassed patients with DFI < 436 and DFI > 1562 days, respectively.

### 4.10. Statistics

Statistical analyses were performed using GraphPad Prism, version 9. Differences between groups of samples were determined by an unpaired *t*-test after testing whether the values fit the criteria for a normal distribution (tests applied: Shapiro–Wilk test and Kolmogorov–Smirnov test). Pearson’s correlation analyses were performed if data were normally distributed. For data with non-normal distributions, Spearman’s correlation analyses were performed.

## 5. Conclusions

Our findings provide evidence that CTCF restrains *IL6* expression by interacting with the *IL6* promoter, a form of regulation disrupted in highly tumorigenic cells and perhaps in therapy-resistant tumors. The effect of CTCF on *IL6* transcriptional regulation in patient-derived samples should be corroborated in future experiments. In those assays, it will be important to consider the potential contribution of other well-known transcriptional activators of the IL6 gene, such as AP1 [29], NF-kB [65], or NFAT [29].

## Data Availability

Data is contained within the article.

## Abbreviations

The following abbreviation are used in this manuscript

Tam-Res	Tamoxifen-resistant MCF7 cells
TF	Transcription factor
DFS	Disease-free survival
DFI	Disease-free interval
